# Supplementary material for: Improving Toilet Usability and Cleanliness in Public Schools in the Philippines Using a Packaged Operation and Maintenance Intervention
Source: Int J Environ Res Public Health. 2022 Aug 15;19(16):10059. doi: 10.3390/ijerph191610059 (PMC9407854; doi:10.3390/ijerph191610059)
Supplement: Supplementary file 1 [file ijerph-19-10059-s001.zip › ijerph-1815231-supplementary.pdf]

## Supplementary material

### Sanitation Assessment Tool (SAT)

1. What type of student toilets are at the school? (check one – most common)

☐ Flush or pour-flush toilets
☐ Pit latrines with slab
☐ Pit latrines without slab
☐ Composting toilets
☐ Hanging latrines
☐ Bucket latrines
☐ No toilets or latrines

Please insert tallys:

| | Girls-only toilets | | | Boys-only toilets | | | Shared toilets | | | Urinals | | |
|---|---|---|---|---|---|---|---|---|---|---|---|---|
| 2. How many toilets are at the school? | | | | | | | | | | | | |
| 3. How many toilets are currently usable? (accessible, functional and private) If none, skip question 4 | | | | | | | | | | | | |
| 4. How many of the **usable** toilets are currently clean, somewhat clean and not clean?* | clean | Somewhat clean | Not clean | clean | Somewhat clean | Not clean | clean | Somewhat clean | Not clean | clean | Somewhat clean | Not clean |
| | | | | | | | | | | | | |

5. Are water and soap available at most of the handwashing facilities?

☐ Yes, water and soap
☐ Water only
☐ Soap only
☐ Nor water, nor soap

Further explanations:

Urinals: in the case of trough urinals, count one urinal every 24 inches / 60 cm.

Accessible: not locked from the outside.

Functional: hole is not blocked, water is available for flushing.

Private: a toilet can be locked from the inside.

Clean: The toilet do not have a strong smell or significant numbers of flies or mosquitos, and there is no visible faecal matter in the toilet or around the facility.

Somewhat clean: there is some smell and/or some sign of faecal matter in the toilet
Not clean: there is a strong smell and/or presence of faecal matter in the toilet

**Cleaner Toilets, Brighter Future (CTBF) instrument**

Please complete the below questions per toilet

| | Yes | No |
|---|---|---|
| 1. Does the toilet cubicle have a door? | | |
| 2. If there is a cubicle door, can it be opened (unlocked, or locked with key available)? | | |
| 3. Does the toilet cubicle protect the user from outside views (e.g., no large cracks, holes in wall or broken door)? | | |
| 4. Does the door lock from inside? | | |
| 5. Is the toilet cubicle free of litter? | | |
| 6. Is the toilet bowl intact? | | |
| 7. Is the toilet hole unblocked? | | |
| 8. Is water for flushing available inside the cubicle (e.g., piped water or water bucket with dipper)? | | |
| 9. Is the toilet bowl/pan/slab free of visible traces of feces and urine? | | |
| 10. Are the cubicle walls and floor free of visible traces of feces or urine? | | |

**Manual and video**

Link to the “WASH in Schools Operation & maintenance” manual:
http://www.fitforschool.international/resource/wash-in-schools-operation-maintenance-manual/

Additional information:
For estimation of the cost of basic tools/equipment and consumables needed for proper operation and maintenance of WASH facilities for public schools, exploration of specification and quantity was done using interviews. Fifteen key informant interviews were conducted that involved school and office janitors, school heads and staff, and non-professional cleaners. Fifty experiments were conducted to measure the amount of consumables needed per cleaning activity to inform and calculate the average cost of consumables for the entire school year.

Link to the video about school toilet cleaning and maintenance:
http://www.fitforschool.international/resource/school-toilet-cleaning-and-maintenance/
